# Attendants in the Olden Times

**Published:** 1892-11-19

**Authors:** 


### HOSPITALS FOR THE INSANE.

### ATTENDANTS IN THE OLDEN TIME.

This sounds very ancient, but age is not always measured by the lapse of time. Decisive changes—we might almost call them revolutions—mark time more emphatically, and detach the past from the present with a distinctness clearer than the slow indefinable process of gradual change. The asylum attendant and nurse of to-day can no more be linked with the "keepers" of thirty years ago than light with darkness, or sunshine with shadow. How many of our asylum readers have read Charles Reade's famous novel "Hard Cash"? a novel that on its first appearance roused and agitated the British public with a vehemence of which we have little conception to-day. "Hard Cash" was a novel of a class much maligned at the present time by some critics, "the novel with a purpose"; and it cannot be denied that it excited and enraged the people of this country, and more even than the general public startled the self complacency and terrified the minds of the proprietors of private asylums thirty years ago. Charles Reade marshalled his facts and arguments with telling force and pitiless vengeance, and—told in the form of a tragic romance—his story took hold of the public mind and blazed like wild fire. The columns of the *Daily News* and *Pall Mall Gazette* gave space to a correspondence characterised by strong statements and counter-statements, in which the author of "Hard Cash" again distinguished himself by his trenchant criticism, and drew a graphic picture of horrible cruelties in asylums. All this is ancient history, but it was not without effect at the time. Indeed, it is probable that its effects may still be traced in the readiness of the public to believe evil of asylum officials. The child suffers for the sins of the parent, and the ignominy which still hangs to asylums, shadowy though it comparatively is, may be regarded as an inheritance from the attendants of the olden time.

There is no use denying the fact that before the days of non-restraint, and for long after, asylums were sometimes the abodes of cruelty, injustice, and oppression; but it is equally true that human nature in even those ignorant times was not all brutal, for native benevolence and tact are no special possession of the present time. Charles Reade's crusade was too inflammatory, and the susceptible public caught fire too easily over the matter. Our asylum attendants and nurses who read "Hard Cash"—and everyone should read it—must feel how bitterly the stigma of reproach has been cast on their fellows of the olden time, and how much harm to the service must have resulted by his ill-advised enthusiasm, his too facile pen, and his too ready and sweeping denunciation of a much-enduring class of public servants, because of certain concrete examples of brutality which existed in some asylums.

Size, weight, and muscle were three great qualifications of the "keeper" of the olden time. Sometimes these were associated with good nature and tact, and then a really valuable, though uneducated, attendant was the result, for a big man, if he is wise, will use his muscle as little as possible, and will exhibit in trying circumstances his canny qualities and the utmost good nature. Some of our readers will remember the once famous remark of John Bright, the great statesman and people's tribune, that "force is no remedy." These words were not uttered in connection with asylums, but the text is as good for attendants as John Bright considered it good for the solution of the Irish question, and perhaps better. Restraining force was the quality sought for in the past — in other words, muscular strength and mechanical contrivances. Moral excellence, intelligence, and education are the qualities most called for at the present day. In the olden time the lunatic raged, and the asylums were bear gardens, as we rarely see them in the present day except in uncivilised countries. The patients were not regarded as patients, they were looked on as devils incarnate, impulsive, violent, profane, and filthy, far exceeding anything in our present experience; they were kept almost naked, their fury was that of wild beasts, and many of them were kept in chains. It required a quick eye, a cool head, and a strong arm to hold your own with them. If you could ask an attendant of the olden time what were his instructions, he would stare at you and try to think out a difficult question, for instructions were meagre, and one or two simple ideas, viz., to subdue the patient and prevent escape regulated the whole duty and conduct of the attendant. The old idea was that the attendant must make the patient feel that he was master, and this could only be done by a fight in which the attendant had the better of it, and the patient got a shaking that embittered him against the attendant, and did not always subdue, though it might conquer for a time. It was also considered a sign of weakness for an attendant to call for assistance. A young attendant nev considered that he had won his spurs until he had tackled a violent, aggressive patient single-handed, and mastered him by strength of muscle and bone and sinew. It did not strike them that to restrain with chains, straight waistcoats, locked cells, and instruments of torture was rubbing against the grain and rousing fierce animal passion. They had no conception that there was a human identity behind the mad raving, the restive character, the fierce, glaring eyeballs, the day and night violence, and excitement. That touch of nature which makes the whole world kin was sadly lacking in the asylums of the olden times, and thus the "keepers" acquired a character worse far than that of the rough policeman or prison warder and the poor lunatic suffered accordingly.

Old superintendents of asylums, whose memories reach far into the past, will not accept this as a full and adequate description of the atten dant of the olden time; nor indeedis it. We should be unfair to a class which has been much and virulently abused if we did not acknowledge frankly that our preceding description represents a type of attendants under the old *regime.* But common-sense attendants, whose tact and good sense often then, as now, there was a leaven—a leaven of good humane saved rough handling, and came as balm to the sore-fretted, exasperated lunatic. The following illustration of tact and good sense in an attendant of the olden time speaks for itself: Willie B— was an epileptic idiot whose religious feelings were early fostered by a pious mother; but in paroxysms of excitement, after fits, he was most dangerous. One day in the sudden accession of one of those paroxysms he lifted his spade and—powerful fellow that he was—would have split the skull of anyone who came near him at the moment. It was a crisis of suspense, the attendants hesitating and helpless, when a big, powerful, attendant with a kindly gleam in his eye, stepped quietly to the rescue, took off his cap, and said quietly, "Give us a word of prayer, Willie." The idiot took his cap, began the Lord's Prayer, his face livid and contorted, and his muscles quivering with suppressed passion the while; he concluded the prayer, put on his cap, but the fire was not completely subdued. The wise attendant took off his cap a second time. "Give us another word, Willie." The idiot tried again, the smouldering wrath dying out before the prayer was finished. The attendant was a man of observation; he knew his patient; he was a man of tact, he knew how to navigate him even in turbulent seas. He was one of the leaven of the old school, and the more we have of his quality the better for our asylums.

---

The German tradesman who advertised that he would give ten boxes gratis of his cocoa to anyone who could show him that it is injurious to health must have hailed originally from the Emerald Isle. Applications for the proffered present are not likely to be overwhelming.

